# Modeling the Significance of Motivation on Job Satisfaction and Performance Among the Academicians: The Use of Hybrid Structural Equation Modeling-Artificial Neural Network Analysis

**DOI:** 10.3389/fpsyg.2022.935822

**Published:** 2022-06-20

**Authors:** Suguna Sinniah, Abdullah Al Mamun, Mohd Fairuz Md Salleh, Zafir Khan Mohamed Makhbul, Naeem Hayat

**Affiliations:** ^1^Graduate School of Business, Universiti Kebangsaan Malaysia, Bangi, Malaysia; ^2^Global Entrepreneurship Research and Innovation Centre, University Malaysia Kelantan, Kota Bharu, Malaysia

**Keywords:** higher education, motivation, job satisfaction, academic performance, SEM-ANN analysis

## Abstract

The competition in higher education has increased, while lecturers are involved in multiple assignments that include teaching, research and publication, consultancy, and community services. The demanding nature of academia leads to excessive work load and stress among academicians in higher education. Notably, offering the right motivational mix could lead to job satisfaction and performance. The current study aims to demonstrate the effects of extrinsic and intrinsic motivational factors influencing job satisfaction and job performance among academicians working in Malaysian private higher educational institutions (PHEIs). Cross-sectional data were collected from the Malaysian PHEIs and the randomly selected 343 samples. The data analysis was performed with the dual analysis of partial least square structural equation modeling (PLS-SEM) and artificial neural network (ANN) analysis. As a result, it was found that financial rewards, promotion, performance appraisal, classroom environment, and code of conduct significantly predicted job satisfaction. The code of conduct, autonomy, and self-efficacy strongly influenced job performance. The relationship between job satisfaction and job performance was highly moderated by self-efficacy. It was suggested from the ANN analysis that the three prominent factors influencing job satisfaction are financial rewards, performance appraisal, and code of conduct. The analysis supported three significant factors influencing job performance: self-efficacy, performance appraisal, and code of conduct. The management of PHEIs should build the correct policies to transform job satisfaction into job performance. Self-efficacy plays an essential role in activating job performance. Other significant motivating factors that promote job satisfaction and performance, such as emotional intelligence, mindfulness, and other personal traits, should be included in future studies. In addition, future research could use a mixed-method or multi-respondent approach to investigate the important variables and their impact on lecturers’ job satisfaction and performance.

## Introduction

“Performance” has been an aspect to be discussed and widely studied since the 1980s. It is represented as an “act of doing work or duty” assigned to an individual in a work setting (Speer et al., 2020). Academic performance is viewed as a responsibility and outcome of the effort within their authority level. Performance is measured by several criteria, namely quantity and quality, cost-effectiveness and timeliness, and the need for interpersonal impact, which includes supervision (Munyengabe et al., 2017a). With the growth of the knowledge economy, universities are faced with intensive competition on both domestic and global levels toward rating and performance (Ong et al., 2020). The lecturers’ performance is the key contributor to the academic quality of universities and colleges. Therefore, universities and colleges should empower their human capital to gain practicability, deliver state-of-the-art academic services, and achieve first-class academic status (Milkhatun et al., 2020). It is noteworthy that the complex nature and difficulties in measuring the outcome have become the major drawbacks in assessing individual and institutional performance in higher education (Malek et al., 2020). Currently, academicians face highly competitive and demanding workplaces in higher educational institutions (Kim and Fah, 2020). The academicians are prompted to have higher workloads in the form of classroom teaching and learning duties, engaged in research, publications, grants, training, supervision, invigilation, administrative tasks, and social commitments (Arora, 2020). As a result, lecturers lose interest in their jobs and consider academic work to be less interesting (Chong et al., 2019). Academics who are constantly unhappy and dissatisfied will notice a change in their work execution and nature. Other tasks may satisfy them, but the entire workload does not (Omar et al., 2020). Furthermore, this may result in staff turnover, which can be costly to the institutions (Tentama and Riskiyana, 2020). The institution may experience a loss of personnel investment, staff replacement costs, training costs, and a delayed work process. As a result, assessing and improving motivational factors among academics is necessary to prevent unproductive conduct and improve work performance. Hence, the assessment and improvement in motivational factors among academicians are needed to reduce unproductive behavior and improve work performance among academicians.

Academicians are responsible for shaping young generations who are the assets of the country (Ekundayo and Ayodele, 2019). Low morale and unmotivated performance may negatively influence the students (Chong et al., 2019), which leads to the utmost importance in maintaining the academicians’ motivation. The motivational theory suggests the psychological (intrinsic) and survival (extrinsic) needs (Robbins and Judge, 2009). To be specific, intrinsic motivation is defined as a type of motivation based on the natural interest of individuals in various activities with challenges and uniqueness. It does not involve external rewards, but rather the individuals’ expression regarding themselves and their interests (Ryan and Deci, 2020). In contrast, extrinsic motivation is triggered by external factors that are primarily financial. It is also known as the outcome of the performance of an activity, which includes financial reward (FR), promotion, and performance appraisals (PALs). These factors have been adapted for the study from the literature, with only the three aforementioned factors being selected (Robbins and Judge, 2009). Intrinsic motivation is strongly linked with the natural well-being of the teaching and learning process, which is systematically compromised by common practices among teachers and parents (Ryan and Deci, 2020). However, although extrinsic motivation is on the contrary to intrinsic motivation, self-determination theory (SDT) suggests that some forms of extrinsic motivation are inadequate, while some forms are effective (Matos et al., 2021).

Malaysian higher education offers a unique combination of learning opportunities that attract local and international students. A total of 20 public and approximately 450 private universities and higher education colleges offer eight levels of higher education programs in Malaysia under the Malaysian Qualification Framework (MQF 2.0) (Azman and Abdullah, 2021). Currently, 30% of university students are international students. The Malaysian higher education system offers education to attract reasonable foreign exchange for the country rather than uplifting the social, economic, and political economy (Ministry of Higher Education [MOHE], 2019). Quality teaching is at the heart of the higher education system, and it can only be achieved if higher education faculties are satisfied and well-functioning (Lee et al., 2022). The higher number of private higher educational institutions in Malaysia demonstrates that private investors are progressing toward gaining the lucrative higher education market. The PHEI industry share would have reached USD 0.85 billion by 2021 and USD 1.50 Billion by 2026 (MIDA, 2022). However, quality education needs quality academicians working in a suitable work environment in a motivated manner (Hartinah et al., 2020).

The current study seeks to analyze the motivation leading to job satisfaction and performance among academicians. It also aims to explain the motivational factors contributing to the lecturer’s job satisfaction and performance. A questionnaire-based survey has been employed among 343 academicians working across peninsular Malaysia to address the following research questions; (1) to what extent does the motivation (extrinsic/intrinsic) affect lecturers’ job performance? (2) how does lecturers’ job satisfaction intercede the relationship between motivation (extrinsic/intrinsic) and lecturers’ job performance? (3) how does lecturers’ self-efficacy influence the relationship between their job satisfaction and job performance. Following that, the remaining sections present the pertinent literature, the method adopted for data analysis, and the discussion of the results.

## Literature Review

### Theoretical Foundation

Many theories are perceived as universal for the prediction and understanding of the needs categories that employees attempt to achieve within their motivation and fulfillment as a guide of priority or pre-potency within their work (Mojolou et al., 2018). Motivation is constantly linked with numerous prominent theories, each with different concepts and circumstances that impact performance and satisfaction (Ryan and Deci, 2020). Some of the remarkable theories are Maslow’s Hierarchy of Needs (MHN), Herzberg’s two-factor theory (HTFT), social cognitive theory (SCT), Vroom’s Expectancy theory (VET), and Two-Factor Theory (TFT) (Robbins and Judge, 2009). The paper content theories include Herzberg’s two-factor theory and social cognitive theory. Herzberg’s theory has also evidenced that individuals are not satisfied with the lower workload. However, they gain satisfaction through the achievement of psychological needs, recognition and growth, responsibility, promotion, and the nature of the work (Thant and Chang, 2021).

In parallel, social cognitive theory posits that two cognitions, namely outcome expectancies and self-efficacy, are essential in self-regulation (Schunk and DiBenedetto, 2020). It has been suggested that intrinsic and extrinsic motivations are the most efficient methods of motivating an individual. A correct and effective motivation leads to individual self-efficacy, which is followed by job satisfaction and success in terms of organizational efficiency.

### Hypotheses Development

#### Financial Rewards

Financial rewards refers to monetary incentives that an employee receives in return for the appropriate performance in line with organizational objectives (Kaiser et al., 2020). The FRs denote the types of sessional earnings, bonus pay, pay increment, indirect costs, and additional reimbursement (Malek et al., 2020). Since the 1980s, the professionalism of lecturers has received relatively notable attention for enhancement, mainly by improving their motivation and job satisfaction through the FR system (Isa and Palpanadan, 2020). Koo et al. (2020) added that FR does not only motivate employees, but also increases job satisfaction and performance of employees in an organization. Correspondingly, Pham-Thai et al. (2018) highlighted an increase in employee productivity upon the increase in the pay structure. The recent empirical work demonstrated that FRs positively influence job performance, while the employees are more concerned about the extrinsic reward systems that include salary, bonuses, or commissions that could increase their satisfaction and the organization profits (French et al., 2020; Basalamah and As’ad, 2021). Accordingly, the following hypotheses are suggested:

Hypothesis 1 (H_1_): *FRWs positively affect the lecturer’s job satisfaction (LJS).*
Hypothesis 2 (H_2_): *FRWs positively affect the lecturer’s job performance (LJP*).

#### Promotion

Employee performance compensation is not always cost-effective (Cullen and Perez-Truglia, 2018). Employees respect managerial titles more since they have a formal status and may be put on resumes. The Promotion (PRN) denotes the opportunity offered to an employee based on the upward career movement provided by the employer to the high-performing employees. Promotion is offered in the form of job title, seniority, or pay raise across the board that follows the hierarchy (Asaari et al., 2019). However, the remuneration for motivating workers’ performance would not be as cost-effective (Cullen and Perez-Truglia, 2018). Employees value managerial titles due to their link with promotion, which allows them to consider their status (Benson et al., 2019). Bognanno and Melero (2016) postulated that promotions reveal different dimensions of skills and capabilities of different types of workers. From the academic settings, promotions are perceived as the most perceptible indicator of scholarly academic status. In the education systems, lecturers’ promotions remain essential as they increase academicians’ job satisfaction and performance (Ekundayo and Ayodele, 2019). Hence, the following hypothesis is established:

Hypothesis 3 (H_3_): PRN positively affects the lecturer’s job satisfaction (LJS).
Hypothesis 4 (H_4_): *PRN positively affects the lecturer’s job performance (LJP).*

#### Performance Appraisals

Performance appraisals is utilized with various names, which include performance evaluation, performance review, personnel rating, employee evaluation, and employee appraisal (Speer et al., 2020). Lee et al. (2022) outlined various criteria based on three main groups: teaching, research, and service for the academic professional. A focus was placed on certain education processes, such as input (e.g., staff qualification, nature of students, and material resources), processes (e.g., teaching approaches and student involvement including feedback), and output (e.g., students’ qualifications, rates of employment, and staff publications) (Matos et al., 2021). Despite the lecturers’ tasks and responsibilities, the PAL process is viewed as the guide for the lecturers to improve their teaching ability and put their utmost effort (Al-Ashqar, 2017). Individual performance may now be easily tracked, and feedback is more global than ever before (Parker and Grote, 2020). Companies have started to modernize their performance management systems by implementing advanced tools such as staff monitoring software, workplace tracking devices, feedback-tracking apps, and changing their performance feedback approaches. Mwangi and Njuguna (2019) stated that technology allows managers to communicate and refresh input on a more frequent and flexible basis than traditional approaches, which are reviewed on a monthly, quarterly, and yearly basis. It was emphasized that PAL is an organizational tool to satisfy the employees and increase overall individual and organizational performance. Thus, the following hypothesis is suggested:

Hypothesis 5 (H_5_): *PAL positively affects the lecturer’s job satisfaction (LJS).*
Hypothesis 6 (H_6_): *PAL positively affects the lecturer’s job performance (LJP).*

#### Classroom Environment

Classroom environment (CET) denotes the physical characteristics of the classroom and a combination of the lighting, temperature, and other aspects such as the ventilation system, floor, walls, room size, desks, chairs, rugs, whiteboards, and computers (Wargocki et al., 2020). Universities exert effort to create an attractive classroom environment by building a strong impression and directly influencing lecturers’ perceived teaching quality, which increases the overall performance (Li and Koedel, 2017). Adding safety feathers to the classroom reduces the likelihood of accidents and mishaps, which is essential for maintaining a positive learning environment (Thanajirachot et al., 2019). In the context of the current study, HTFT is pertinent for clarifying that a decent CET contributes to worthiness and satisfaction among educators and students during their teaching and learning processes (Munyengabe et al., 2017b). The HTFT also asserts that a conducive environment motivates lecturers in their daily teaching activities (Wargocki et al., 2020). Both academicians and students expect a conducive, comfortable, and attractive CET to participate in. HTFT does not only accommodate the secondary level or the primary level, but it could also be an essential factor at the university level. Therefore, the following hypotheses are proposed:

Hypothesis 7 (H_7_): *CET positively affects the lecturer’s job satisfaction (LJS).*
Hypothesis 8 (H_8_): *CET positively affects the lecturer’s job performance (LJP).*

#### Code of Conduct

Code of conduct (CCT) refers to principles and rules regulating the social institution actions toward their stakeholders and the stakeholders’ (especially employees) actions toward the institution (Alizadeh et al., 2021). The CCT plays a dominant role in the teaching career (Maxwell, 2020). Teaching comprises an exclusive set of ethical ideas and professional values that describe the ethical responsibility of conduct, which include due process, intellectual honesty, integrity, respect for privacy and dignity, and personal achievement (Schwimmer and Maxwell, 2017). In the academic context, most of the research works revealed that the CCT offers guidelines for college presidents, institutional advancement officers, academic officers, and individual college and faculty members of the university on how to perform their respective roles (Maxwell, 2020). According to Munyengabe et al. (2017b), Rwandan academics are dedicated to their jobs, but they do not avoid using CCT to improve job satisfaction and performance. Hence, the following hypotheses are proposed:

Hypothesis 9 (H_9_): *CCT positively affects the lecturer’s job satisfaction (LJP).*
Hypothesis 10 (H_10_): *CCT positively affects the Lecturer’s Job performance (LJP).*

#### Autonomy

Autonomy (ATM) is defined as the perception of independence to use personal and professional competence at work (Pearson and Hall, 1993). Some teachers view autonomy as their freedom to develop their academic qualifications for managing the classroom, while others view autonomy as freedom from obstruction and control (Muhammad et al., 2020). The HTFT positions the practices of autonomy with a sense of responsibility and accountability, which contributes to excellence in the institution academic, government, and finance (Saragih, 2017). Self-empowerment among lecturers has a considerable impact on intrinsic regulation as compared to self-determined regulation. Controlled regulation, on the other hand, is unaffected (Schunk and DiBenedetto, 2020). Due to its impact on lecturers’ overall performance and happiness, self-directed behavior among professors should be prioritized. Therefore, the following hypotheses are suggested.

Hypothesis 11 (H_11_): *ATM positively affects the lecturer’s job satisfaction (LJS).*
Hypothesis 12 (H_12_): *ATM positively affects the lecturer’s job performance (LJP).*

#### The Effect of Job Satisfaction on Job Performance

Lecturer job satisfaction presents the employees’ actual perception of the job and could exhibit the real performance at the workplace. The literature supported the argument highlighting that employees are more productive and able to play a significant role in higher organizational effectiveness (Che Nawi et al., 2016; Jehanzeb and Mohanty, 2019; Jermsittiparsert et al., 2019). However, contradictory evidence has presented that job satisfaction may not necessarily lead to job performance (French et al., 2020). It would be noteworthy to explore the impact of job satisfaction on the lecturers’ job performance in higher education. Therefore, the following hypotheses are suggested:

Hypothesis 13 (H_13_): *LJS positively affects the lecturer’s job performance (LJP).*

#### The Mediating Role of Lecturer Job Satisfaction

Lecturer job satisfaction estimates the feelings and attitudes of employees toward their job (Mojolou et al., 2018). It also depicts the emotional state of pleasure as a result of the judgment over individuals’ jobs as achieving or enabling the achievement of their values (Pham-Thai et al., 2018). However, job satisfaction among lecturers is dynamically significant in impacting the success of a university vision and mission (Che Nawi et al., 2016). Extrinsic motivation (FRW, PRN, PAL) and intrinsic motivation (CET, CCT, ATM) have been demonstrated as the stimulus for overall job satisfaction (Asaari et al., 2019; French et al., 2020). Based on the above discussion, the following hypotheses are proposed:

HM1–HM3: *The relationship between intrinsic motivation (FRW, RPN, PAL) and job performance is significantly mediated by job satisfaction.*
HM4–HM6: *The relationship between extrinsic motivation (CET, CCT, ATM) and job performance is significantly mediated by job satisfaction.*

#### The Moderating Role of Self-Efficacy

Self-efficacy denotes individuals’ innate understanding of their capability and functions as an activation force to engage in a particular situation. In the academic context, lecturers with high self-efficacy could gain achievement, use creative teaching techniques, and create a perfect classroom environment for better performance (Kim and Fah, 2020). Self-efficacy empowers the lecturer to believe in themselves, offer organized teaching efforts, and confidently engage in superior work performance (Lee et al., 2022). Due to their influence on how lecturers comprehend their core duties and enhance their planning and organizing abilities related to such consequences, these aspects are critical for work success (Matos et al., 2021). This is in line with the assumptions of cognitive theory by Albert Bandura (Dellinger et al., 2008), who believes that self-efficacy is an essential characteristic of common causation because of the impact these beliefs have on the accomplishment of tasks and related goals. Bandura (1977) stated that self-efficacy could be changed according to the situation and it varies depending on the context and task. From the lecturers’ point of view, self-efficacy certainty is defined as individuals’ beliefs in their capabilities to execute their teaching tasks effectively and efficiently. Based on the discussion, the following hypotheses are suggested:

H14: *SEY positively affects the lecturer’s job performance (LJP).*
HM7: *The relationship between the lecturer’s job satisfaction and job performance is significantly moderated by self-efficacy.*

**FIGURE 1:**
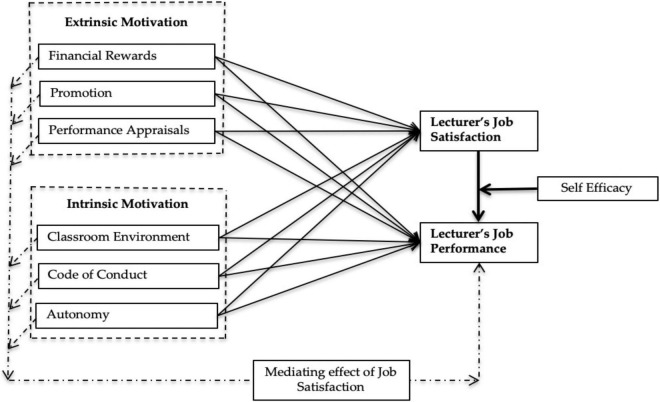
Research framework.

All the associations hypothesised in the above section are presented in Figure 1.

## Research Methodology

The current study assumed an explanatory study design established on the deductive method. A cross-sectional survey-based strategy was utilized for the current study, followed by data collection to explore job satisfaction and performance among the Malaysian lecturers working in PHEI. The target population of this study’s interest comprised the academicians from Private Universities throughout Peninsular Malaysia (see Supplementary Appendix 1). Despite the fact that there are more colleges in Malaysia than universities, the focus of this study is on university lecturers; the primary rationale for this choice was the disparity in job criteria between colleges and universities. The job descriptions highlighted the differences in total teaching hours per week and the diverse roles and duties of lecturers (e.g., research, publication, consultancy, administration tasks, and academic service-related activities). Despite the fact that college and university instructors are often academic equals, university lecturers’ workloads and academic service-related activities are relatively greater than those of college lecturers. Based on the following table, Malaysia’s total PHEIs comprised 448 entities comprising 51 universities, 10 international branch campuses, 38 college universities, and 349 colleges (Ministry of Higher Education [MOHE], 2019). Therefore, to generalize the population of PHEIs’ lecturers based on the above justification, the universities in Peninsular Malaysia (51 entities, excluding 1 university from Sabah) were selected as the representative population sample of the overall PHEIs population. The Ministry of Higher Education Malaysia [MOHE] (2018) and Jabatan Pendidikan Tinggi (2019) directories were used to compile a list of universities for this study. As demonstrated in Supplementary Appendix 2, the content in both guides is divided into categories based on the types of campuses and operations. Non-probability sampling with a purposive sample technique was used to select the total number of respondents.

### Calculation of Sample Size

Due to the unavailability of PHEIs’ lecturer directories as a whole, the overall population of PHEIs’ lecturers in Malaysia was assumed to be 1,000,000 and above. As indicated through Krejcie and Morgan’s sample size, a sample size of 384 was advocated for an assigned population of 1,000,000. Hence, the sample size of this study was indicated with a minimum of 384 lecturers from PHEIs in Malaysia. However, this figure was rounded up to 400 respondents for ease of analysis purposes. Next, the sample size for the current work was estimated using G-Power 3.1 with a power of 0.95 and an effect size of 0.15. The mandatory sample size for the model amounted to 160 with eight input variables (Faul et al., 2007). PLS-SEM required a minimum threshold of 200 samples (Chin, 2010). Data collection was conducted using an online survey from the Malaysian PHEIs lecturers with qualifying queries and acquirement of the respondents’ consent. This process took place from September 2021 to November 2021. The final analysis was conducted with 343 valid responses.

### Research Instrument

The survey instrument was developed from previous research with minimal changes to fit the study’s setting and scope. FRs perception was estimated with six items derived from Chiang and Jang (2008), while the perception of the promotion was obtained with six questionnaire items adopted from Munyengabe et al. (2017b), the perception of performance appraisal was evaluated with six statements from the work by Al-Ashqar (2017), classroom environment perception was estimated with six items taken from Munyengabe et al. (2017b), the perception about the CCT was identified with six question items borrowed from the work by Munyengabe et al. (2017b), and perception of autonomy evaluated with the six statements from the work of Johari et al. (2018). The job satisfaction among the respondents was estimated with seven items taken from Munyengabe et al. (2017b), self-efficacy was gauged with the six question items borrowed from work by Sharp et al. (2013), and the job performance was evaluated with the six statements from the work by Sukirno and Siengthai (2011). The question items were measured with the five-point Likert scale. The questionnaire items for the current work with the source are presented in Supplementary Appendix 3.

### Common Method Bias

The single factor accounted for 37.18%, which was below the suggested threshold of 50%. Therefore, the insignificant influence of CMB in the current study was approved (Podsakoff et al., 2012). Another CMB test was conducted to estimate the latent factor correlation for the current model. As a result, the correlation among the latent factors was less than 0.90, which indicated the absence of the issue of CMB in the current work. The CMB evaluated the current study by testing the full collinearity of all study constructs (Kock, 2015). All the study constructs were regressed on the common variable. Variance inflation factor (VIF) values are presented in Table 1. Overall, all VIF values were less than 5.5, which demonstrated the absence of bias in the data collected from a single source (Kock, 2015).

**TABLE 1:** Full collinearity test.

FRW	PRN	PAL	CET	CCT	ATM	LJS	SEY	LJP
2.909	3.230	2.214	1.869	2.067	1.591	3.673	2.574	2.636

*FRW, Financial Rewards; PRN, Promotion; PAL, Performance Appraisals; CET, Classroom Environment; CCT, Code of Conduct; ATM, Autonomy; LJS, Job Satisfaction; SEY, Self-Efficacy; LJP, Job Performance.*

### Multivariate Normality

Multivariate normality for the study data was assessed with the Web Power online tool^1^. Based on the calculated Mardia’s multivariate *p*-value, the study data showed a non-normality issue as the *p*-values were below 0.05 (Mardia’s multivariate skewness = 37275, *p* = 0.00; and kurtosis = 43.320, *p* = 0.00) (Cain et al., 2017). Following the multivariate non-normality, the current work employed the partial least square–structural equation modeling (PLS-SEM).

### Data Analysis Method

The current study employed the PLS-SEM procedure to confirm the projected model and assess the proposed hypotheses using the SmartPLS 3.2 tool. The PLS-SEM is suitable to be used with a small data set and to reveal the casual-predictive association between the model variables (Kock, 2015). The path model hypothesis was tested with path beta (coefficient), confidence interval, *t*-values, and *p*-values (Hair et al., 2019).

#### Artificial Neural Network Analysis

Artificial neural network analysis is a pseudo-investigative method encompassing three layers: input, output, and hidden (Hayat et al., 2021). The input and output neurons are linked *via* the veiled layer (Gbongali et al., 2019). The hidden layer functions in the same manner as the human brain block-box (Hayat et al., 2020, 2021). The ANN analysis is a non-compensatory diagnostic method that uses a deep learning method with three layers: input, output, and hidden (Gbongali et al., 2019). The information is separated into three categories, namely training, testing, and holding out the sample (Hayat et al., 2021).

The predictive score for the model was calculated through the comparison of the Root Mean Square Errors (RSME) for the training and testing of the model (Hayat et al., 2020). The minor difference between the RSME scores during the training and testing of the model demonstrated the high predictive and difference of the RMSE scores between the training and testing of the model, including the low predictive accuracy (Gbongali et al., 2019). The estimation of normalized relevance for the model latent factors was identified through the sensitivity analysis (Hayat et al., 2021). The following formula was applied in the study to gain the goodness-of-fit index:


$$R^{2}=1-\frac{R\,M\,S\,E}{S\,S\,E}.$$

## Data Analysis

### Demographic Characteristics

In the current study, 63.0% of the respondents were female. While 5% of the respondents were Bachelor’s degree holders, 61.5% of the respondents obtained the Master’s degree, and the remaining respondents obtained the Doctor’s degree. Most of the respondents (39.9%) were 26–35 years old, 35.6% of the respondents aged between 36 and 45 years old, 16.6% of the respondents aged between 46 and 55 years old, 6.4% of the respondents aged over 55 years old, and the rest of the respondents were under 25 years old. Moreover, 75.8% of the respondents held the permanent position, while the remaining respondents had the contract position. Following that, 42.0% of the respondents received less than 5 years of working experience, 33.2% of the respondents gained 5–10 years of working experience, 14% of the respondents had 11–15 years of work experience, 8.7% of the respondents gained 16–20 years of work experience, and the remaining respondents gained over 20 years of working experience. A total of 51.3% of the respondents carried the lecturer position, 18.4% of the respondents held the senior lecturer position, 15.2% of the respondents held the senior lecturer position with a PhD, 5.8% of them carried the associate professor position, 1.7% held the tutor position, and only 0.9% of the respondents had assistant professor position. The results are shown in Table 2.

**TABLE 2:** Demographic characteristics of respondents.

	*N*	%		*N*	%
*Gender*			***Education***		
Male	127	37.0	Bachelor’s degree or equivalent	17	5.0
Female	216	63.0	Master’s degree	211	61.5
Total	343	100.0	Doctoral degree	115	33.5
			Total	343	100.0
*Age Group*					
Below 25 years	5	1.5	*Employment Status*		
26 – 35 years	137	39.9	Contract	83	24.2
36 – 45 years	122	35.6	Permanent	260	75.8
46 – 55 years	57	16.6	Total	343	100.0
More than 55 years	22	6.4			
Total	343	100.0	Academic Position		
			Tutor	6	1.7
*Experience*			Lecturer	176	51.3
Below 5 years	144	42.0	Senior Lecturer	63	18.4
5 – 10 years	114	33.2	Senior Lecturer with Ph.D.	52	15.2
11 – 15 years	48	14.0	Assistant Professor	3	0.9
16 – 20 years	30	8.7	Associate Professor	20	5.8
More than 20 years	7	2.0	Others	23	6.7
Total	343	100.0	Total	343	100.0

### Reliability and Validity

The suggestions by Hair et al. (2019) and the accomplished latent construct reliabilities were assumed and assessed with the Cronbach’s alpha (CA), DG rho, and composite reliability (CR). The results are presented in Table 3. The CA values for each construct were above the minimum value of 0.70, while the minimum value of the acquired CA score amounted to 0.704 (Henseler et al., 2017). Furthermore, all the DG rho scores of each construct were above the threshold of 0.70, where the lowest value of DG rho was 0.708 (Hair et al., 2019). The CR scores exceeded 0.70, while the lowest CR value was 0.818 (Chin, 2010). The average value extracted (AVE) for all items for each construct should be above 0.50 to justify the suitable convergent validity and withstand the uni-dimensionality of every construct (Hair et al., 2019). Based on the items, the constructs had acceptable convergent validity (see Table 4). All the value inflation factor (VIF) scores of each construct were less than 5.5, which indicated that the issue of multicollinearity was not present in the current model (Kock, 2015).

**TABLE 3:** Reliability and validity results.

	No. of items	Mean	Standard deviation	Cronbach’s alpha	Dijkstra-Hensele’s *rho*	Composite reliability	Average variance extracted	Variance inflation factor
FRW	6	3.067	0.903	0.896	0.899	0.920	0.659	2.901
PRN	6	3.043	0.935	0.911	0.915	0.931	0.694	3.272
PAL	6	3.514	0.788	0.835	0.852	0.881	0.558	2.242
CET	4	4.004	0.657	0.716	0.730	0.824	0.542	2.072
CCT	4	3.867	0.624	0.704	0.708	0.818	0.529	2.090
ATM	6	3.807	0.755	0.924	0.927	0.940	0.724	1.561
LJS	7	3.389	0.786	0.870	0.875	0.901	0.566	3.642
SEY	6	4.066	0.680	0.902	0.923	0.925	0.675	2.262
LJP	6	4.146	0.606	0.913	0.920	0.933	0.699	–

*FRW, Financial Rewards; PRN, Promotion; PAL, Performance Appraisals; CET, Classroom Environment; CCT, Code of Conduct; ATM, Autonomy; LJS, Job Satisfaction; SEY, Self-Efficacy; LJP, Job Performance.*

*Source: Author’s data analysis.*

**TABLE 4:** Discriminant validity results.

	FRW	PRN	PAL	CET	CCT	ATM	LJS	SEY	LJP
**Fornell-Larcker Criterion**									
FRW	0.812								
PRN	0.761	0.833							
PAL	0.636	0.687	0.747						
CET	0.259	0.209	0.310	0.736					
CCT	0.407	0.419	0.438	0.565	0.728				
ATM	0.368	0.393	0.424	0.435	0.510	0.851			
LJS	0.742	0.744	0.677	0.418	0.588	0.484	0.752		
SEY	0.251	0.212	0.317	0.663	0.549	0.440	0.422	0.822	
LJP	0.265	0.279	0.357	0.604	0.561	0.497	0.439	0.776	0.836
**Heterotrait-Monotrait Ratio (HTMT)**									
FRW	–								
PRN	0.839	–							
PAL	0.728	0.777	–						
CET	0.328	0.263	0.409	–					
CCT	0.510	0.524	0.579	0.791	–				
ATM	0.403	0.423	0.477	0.531	0.632	–			
LJS	0.835	0.831	0.788	0.537	0.747	0.535	–		
SEY	0.288	0.236	0.379	0.794	0.680	0.474	0.486	–	
LJP	0.297	0.306	0.416	0.737	0.698	0.537	0.499	0.838	–

*FRW, Financial Rewards; PRN, Promotion; PAL, Performance Appraisals; CET, Classroom Environment; CCT, Code of Conduct; ATM, Autonomy; LJS, Job Satisfaction; SEY, Self-Efficacy; LJP, Job Performance.*

*Source: Author’s data analysis.*

The discriminant validities were assessed with the Fornell and Larcker, 1981, Heterotrait-Monotrait (HTMT) ratio, and loading and cross-loading. Fornell-Larcker criterion results suggested that the current model square root of AVE for a specific construct was more significant than the correlation between the other constructs and confirmed the discriminant validity (Hair et al., 2019). The HTMT ratio values for the study constructs showed satisfactory scores of lower than 0.900, which depicted an acceptable discriminant validity (see Table 2). The item loading and cross-loading enabled the appropriate level of discriminant validity for study constructs (see Supplementary Appendix 4).

### Hypothesis Testing

The model measurement assessment was conducted to examine the study hypotheses. The adjusted *r*^2^ value for the six exogenous constructs (e.g., FRs, promotion, performance appraisal, classroom environment, CCT, and autonomy) on the job satisfaction elucidated 70.2% of the variance in the individual job satisfaction. The predictive relevance (*Q*^2^) score for the part of the model amounted to 0.399, which represented a large predictive relevance (Hair et al., 2019). The adjusted *r*^2^ value for the job performance (e.g., FRs, promotion, performance appraisal, classroom environment, CCT, autonomy, self-efficacy, and job satisfaction) on the job satisfaction amounted to 65.4. The predictive relevance (*Q*^2^) score for the fragment of the model was 0.433, which indicated a large predictive relevance (Hair et al., 2019).

The model standardized path values, t-values, and significance levels are presented in Table 5. The path coefficient between FRW and LJS represents a significant and positive effect of the FRs on job satisfaction. The result offered considerable statistical sustenance to accept the H1. Furthermore, the path value between the PRN and LJS indicated that the promotion positively and significantly created job satisfaction, which provided the statistical support to accept H3. The path between PAL and LJS, which demonstrated the influence of the performance appraisal on job satisfaction, was positive and significant. Thus, the support to accept the H5 was offered. The path coefficient between the CET and LJS signified the classroom environment positive and significant impact on job satisfaction. Overall, the result presented the support to accept H7. The path from CCT and LJS demonstrated a positive and significant impact of the CCT on job satisfaction, which created the support to accept the H9. Following that, the path between ATM and LJS indicated a positive but insignificant impact of autonomy on job satisfaction, which created no statistical provision to accept the H11.

**TABLE 5:** Path analysis.

Hypo		Beta	*T*	*P*	*r* ^2^	*f* ^2^	Q^2^	Decision
***Factors affecting Job Satisfaction***								
H_1_	FRW → LJS	0.306	5.550	0.000		0.132		Supported
H_3_	PRN → LJS	0.287	5.080	0.000		0.100		Supported
H_5_	PAL → LJS	0.146	2.583	0.005	0.722	0.035	0.399	Supported
H_7_	CET → LJS	0.100	2.359	0.009		0.023		Supported
H_9_	CCT → LJS	0.196	4.390	0.000		0.075		Supported
H_11_	ATM → LJS	0.053	1.539	0.062		0.007		Reject
***Factors affecting Job Performance***								
H_2_	FRW → LJP	–0.083	1.367	0.086		0.007		Reject
H_4_	PRN → LJP	0.071	1.173	0.121		0.005		Reject
H_6_	PAL → LJP	0.032	0.699	0.243		0.001		Reject
H_8_	CET → LJP	0.070	1.374	0.085	0.662	0.007	0.433	Reject
H_10_	CCT → LJP	0.119	2.558	0.005		0.020		Supported
H_12_	ATM → LJP	0.125	3.278	0.001		0.030		Supported
H_13_	LJS → LJP	0.023	0.343	0.366		0.000		Reject
H_14_	SEY → LJP	0.542	9.259	0.000		0.384		Supported
***Mediating Effect of Job Satisfaction***								
HM1	FRW → LJS → LJP	0.007	0.327	0.372				No Mediation
HM2	PRN → LJS → LJP	0.007	0.347	0.364				No Mediation
HM3	PAL → LJS → LJP	0.003	0.323	0.373				No Mediation
HM4	CET → LJS → LJP	0.002	0.299	0.383				No Mediation
HM5	CCT → LJS → LJP	0.004	0.337	0.368				No Mediation
HM6	ATM → LJS → LJP	0.001	0.258	0.398				No Mediation
***Moderating Effect of Self-Efficacy***								
HM7	LJS*SEY LJP	–0.076	2.964	0.002			Supported	

*FRW, Financial Rewards; PRN, Promotion; PAL, Performance Appraisals; CET, Classroom Environment; CCT, Code of Conduct; ATM, Autonomy; LJS, Job Satisfaction; SEY, Self-Efficacy; LJP, Job Performance.*

The path coefficient between FRW and LJP represented a significant and positive effect of FRs on job performance. The result offered substantial statistical support for not accepting the H2. The path value between the PRN and LJP demonstrated that promotion positively and significantly encouraged job performance, leading to the statistical provision for not accepting H4. The path between PAL and LJP demonstrated the positive but insignificant influence of the performance appraisal on the job performance, leading to the support for not accepting H6. The path coefficient between the CET and LJP indicated the positive but insignificant impact of the classroom environment on job performance, leading to the absence of support for accepting the H8. The path from CCT and LJP demonstrated a positive and significant impact of the CCT on the job performance, which offered the sustenance to accept the H10. Moreover, the path between ATM and LJP demonstrated a positive and significant impact of autonomy on job performance, which created statistical support to accept the H12. The path from LJS and LJP presented a positive but insignificant effect of job satisfaction on job performance, which offered the support to not accept the H13. Lastly, the path from SEY and LJP showed a positive and significant impact on self-efficacy on the job performance, leading to the acceptance of H14.

### Mediating Analysis of Job Satisfaction

The mediational analysis for the study demonstrated that the relationship between the FRW and LJP was insignificantly mediated by the LJS, leading to no support to accept HM1. Following that, the relationship between the PRN and LJP was insignificantly mediated by LJS, showing no sustenance to accept HM2. The following mediating hypothesis evaluated the relationship between the PAL and LJP, which was mediated by the LJS. However, the analysis presented no support to accept the mediation of LJS between the PAL and LJP and not to accept HM3. Moreover, the relationship between the CET and LJP was insignificantly mediated by the LJS, which showed no support for accepting HM4. The association between CCT and LJP was insignificantly mediated by LJS and offered no sustenance for accepting HM5. Then, the mediating hypothesis evaluated the relationship between the ATM and LJP, which was mediated by LJS. Overall, the analysis showed no support to declare the mediation of LJS between the ATM and LJP and not to accept HM6.

### Moderation Analysis

The moderation analysis result demonstrated that the relationship between the LJS and LJP was significantly moderated by self-efficacy and offered evidence to admit the HM7.

### Multi-Group Analysis

The study assessed the measurement invariance using the measurement invariance of composite models (MICOM) procedure for two groups (Group 1. Work Experience ≤ 10 years, and Group 2. Work Experience > 10 years). The permutation *p*-values for all variables exceeded 0.05, which confirmed the partial measurement invariance. Therefore, the study was able to compare the path coefficients between two groups using PLS-MGA. The results of the two groups (see Table 6) based on work experience showed no significant differences in all associations hypothesized in this study, except for the effect of promotion on job performance. The effect of promotion on job performance among academicians with working experience of 10 years or less was positive and statistically significant. However, the job performance among academicians with working experience of 10 years or longer was negative and statistically significant. The difference between academicians with working experience of 10 years or more and less was also statistically significant. Overall, the results indicated that the promotion had a more significant impact on performance among young academicians compared to senior academicians.

**TABLE 6:** Multi-group analysis.

	Experience ≤ 10 Years (*N* = 258)		Experience > 10 Years (*N* = 85)		Difference		
			
Path	Beta	*p*-value	Beta	*p*-value	Beta	*p*-value	Result
FRW → LJS	0.283	0.000	0.329	0.000	–0.046	0.336	No difference
PRN → LJS	0.280	0.000	0.353	0.000	–0.073	0.297	No difference
PAL → LJS	0.175	0.002	0.030	0.002	0.144	0.121	No difference
CET → LJS	0.089	0.035	0.143	0.035	–0.053	0.261	No difference
CCT → LJS	0.198	0.000	0.172	0.000	0.027	0.380	No difference
ATM → LJS	0.049	0.124	0.116	0.124	–0.067	0.211	No difference
FRW → LJP	–0.117	0.050	0.015	0.050–	–0.132	0.159	No difference
PRN → LJP	0.122	0.036	–0.122	0.036	0.244	0.025	No difference
PAL → LJP	0.036	0.276	0.031	0.276	0.005	0.476	No difference
CET → LJP	0.095	0.064	–0.009	0.064	0.105	0.150	No difference
CCT → LJP	0.116	0.025	0.191	0.025	–0.076	0.256	No difference
LJS → LJP	0.144	0.002	0.034	0.002	0.111	0.105	No difference
ATM → LJP	–0.015	0.426	0.214	0.426	–0.228	0.094	No difference
SEY → LJP	0.529	0.000	0.507	0.000	0.022	0.462	No difference
LJS*SEY → LJP	–0.072	0.023	–0.071	0.023	–0.001	0.481	No difference

*FRW, Financial Rewards; PRN, Promotion; PAL, Performance Appraisals; CET, Classroom Environment; CCT, Code of Conduct; ATM, Autonomy; LJS, Job Satisfaction; SEY, Self-Efficacy; LJP, Job Performance.*

*Source: Author’s data analysis.*

### Artificial Neural Network Analysis (Model 1 and 2)

The multi-layer perception (MLP) ANN was employed for the current work, which involved three layers: input, hidden, and output (Gbongali et al., 2019). The feed-forward-back propagation (FFBP) MLP ANN was employed for the study. The tenfold ANN model in the SPSS neural network algorithm was determined to curtail the overestimated issue of the ANN (Hayat et al., 2021). While 70% of the data was utilized for training, 30% was utilized for testing as per Gbongali et al.’s (2019) suggestion.

The prediction accuracy was evaluated with the RMSE score of the model (Gbongali et al., 2019). As shown in Table 7, the results exhibited high predictive accuracy as the RMSE values of training and testing segments of data, which were close to each other.

**TABLE 7:** RMSE values of artificial neural networks (*N* = 304).

	Sample size (Testing)	Sample size (Testing)	RMSE (Training)	RMSE (Testing)	SSE (Testing)	Sample size (Training)	Sample size (Testing)	RMSE (Training)	RMSE (Testing)	SSE (Testing)
	
*Model A: Factors effecting LJS*						*Model B: Factors effecting LJP*				
1	272	130	0.270	0.350	19.431	293	109	0.475	0.403	16.733
2	292	110	0.295	0.294	12.741	277	125	0.464	0.567	27.377
3	272	130	0.299	0.276	17.659	281	121	0.425	0.461	34.010
4	270	132	0.308	0.222	17.522	291	111	0.390	0.459	22.091
5	276	126	0.313	0.258	17.708	274	128	0.496	0.388	32.714
6	289	113	0.288	0.281	14.738	277	125	0.387	0.426	31.436
7	277	125	0.301	0.254	19.753	280	122	0.431	0.398	36.574
8	281	121	0.304	0.227	16.895	266	136	0.507	0.569	25.735
9	292	110	0.292	0.263	13.962	284	118	0.436	0.365	24.428
10	278	124	0.251	0.301	18.411	274	128	0.389	0.379	29.165
	Mean		0.292	0.272	16.882	Mean		0.440	0.441	28.026
	Standard deviation		0.017	0.035	2.214	Standard deviation		0.042	0.0699	5.702

*Source: Author’s data analysis.*

The relative values of RMSE for training and testing Model A and Model B demonstrated that the data achieved higher predictive accuracy (Hayat et al., 2020). Model A was able to predict the intention to use the MWD by 98.3% through the goodness of fit. In Model B, the goodness of fit amounted to 98.4%, with the intention to use MWD being the most significant contributing factor for the use of MWD. The evaluations are presented in Table 7.

Sensitivity analysis (see Table 8) was employed to evaluate the impact of each input variable in model A to develop job satisfaction for the lecturer (Gbongali et al., 2019). The normalized importance scores for every input construct are gauged with the percentage fraction of the relative importance of every input neuron divided by the highest relative importance (Hayat et al., 2020). As a result, five most significant contributing factors for job satisfaction were FRW, PRN, CCT, CET, and PAL, while the five most contributing factors for Model B included SEY, CCT, PRN, ATM, and LJS.

**TABLE 8:** Sensitivity analysis.

Network	FRW	PRN	PAL	CET	CCT	ATM		
	***Factors affecting LJS***							
1	0.257	0.158	0.170	0.126	0.213	0.077		
2	0.253	0.187	0.121	0.193	0.156	0.090		
3	0.260	0.223	0.129	0.123	0.174	0.090		
4	0.204	0.282	0.099	0.135	0.141	0.139		
5	0.282	0.173	0.092	0.166	0.174	0.113		
6	0.242	0.208	0.148	0.164	0.171	0.068		
7	0.293	0.148	0.156	0.175	0.171	0.058		
8	0.203	0.283	0.114	0.118	0.198	0.084		
9	0.257	0.200	0.120	0.182	0.148	0.093		
10	0.262	0.248	0.121	0.145	0.117	0.106		
Mean Importance	0.251	0.211	0.127	0.152	0.166	0.0918		
Relative Importance	100	83.96	50.53	60.76	66.17	36.53		

***Factors affecting LJS***	**FRW**	**PRN**	**PAL**	**CET**	**CCT**	**ATM**	**LJS**	**SEY**

1	0.076	0.058	0.075	0.081	0.105	0.105	0.065	0.434
2	0.024	0.099	0.091	0.068	0.142	0.093	0.038	0.444
3	0.073	0.44	0.059	0.070	0.110	0.063	0.109	0.474
4	0.058	0.061	0.061	0.070	0.221	0.108	0.048	0.373
5	0.057	0.064	0.073	0.059	0.153	0.107	0.080	0.405
6	0.070	0.073	0.058	0.059	0.108	0.087	0.088	0.458
7	0.090	0.059	0.071	0.070	0.119	0.115	0.040	0.438
8	0.070	0.092	0.083	0.078	0.116	0.098	0.093	0.370
9	0.073	0.035	0.035	0.044	0.176	0.108	0.035	0.494
10	0.053	0.035	0.037	0.054	0.191	0.105	0.070	0.456
Mean Importance	0.064	0.101	0.064	0.065	0.144	0.098	0.066	0.434
Relative Importance	14.81	23.37	14.79	15.02	33.15	22.75	15.32	100

*FRW, Financial Rewards; PRN, Promotion; PAL, Performance Appraisals; CET, Classroom Environment; CCT, Code of Conduct; ATM, Autonomy; LJS, Job Satisfaction; SEY, Self-Efficacy; LJP, Job Performance.*

*Source: Author’s data analysis.*

## Discussion and Conclusion

The findings revealed that financial incentives, promotions, and performance evaluations had no impact on the lecturer’s job performance. The current outcome was consistent with Ong et al. (2020) finding that FRs aided professors’ financial needs while also fostering job happiness. However, this contradicts Koo et al. (2020) finding that financial incentives motivate employees to perform better. Similarly, the findings supported Benson et al. (2019) assertion that career advancement empowers people and fosters a sense of success, which leads to workplace satisfaction. According to Benson et al. (2019), promotion is a great approach for academics to achieve job satisfaction. This is challenged by the findings of Ekundayo and Ayodele (2019), who found that providing promotions may not be the best way to improve academicians’ job performance. Promotion may no longer be a viable approach for achieving job success in academic environments. This research also suggested that performance appraisals are beneficial for promoting job satisfaction rather than job performance. The current study’s findings add to the empirical evidence that Herzberg’s two-factor theory of job satisfaction is applicable to boosting job satisfaction among PHEIs professors. Although extrinsic and hygienic elements are important in predicting job happiness, they are not appropriate for predicting job performance.

However, this present study suggests that intrinsic rewards, classroom environment, and CCT significantly impacted the lecturer’s job satisfaction. The current study sought agreement with the work by Basalamah and As’ad (2021) that the classroom facilitated the delivery of teaching instruction while the academic felt empowered and satisfied. However, the study’s results emphasized that the classroom environment was insignificantly related to the lecturer’s job performance. The current result was not in line with Wargocki et al. (2020). Professional conduct can be improved with the right resources available in academic settings. However, a CCT empowers the job routines as a structure that simplifies the job duties and sequences that create the lecturer’s job satisfaction, which is in line with the statement by Alizadeh et al. (2020). The CCT also offers detailed work customs and requirements established to simplify job performance. It was also confirmed that the code of behavior contributes to job performance. The current result was in line with the consequence established by Maxwell (2020) that the CCT empowered the lecturer’s job performance. Contrary, autonomy insignificantly facilitates job satisfaction, which is not in line with the statement posted by Muhammad et al. (2020) that autonomy is not vital for job satisfaction. However, the study demonstrated that autonomy empowers the lecturers mentally and promotes a sense of job performance. The conclusion of this study was in line with the result presented by Muhammad et al. (2020), that the perception of autonomy was related to the employees’ job performance. The lecturers are knowledge workers and require autonomy as an empowerment tool. The perception of autonomy offers the control that leads to the sense of personal responsibility and accountability, which controls the perception of self-control and regulation.

The current study added to the literature that self-efficacy plays a significant role in exhibiting job performance and transforming job satisfaction into job performance. The recent research also adds to the literature establishing self-efficacy as a social-cognitive force that facilitates job performance. Moreover, the current work offers the practical implications that higher educational intuitions are required to harness self-efficacy, which allows the achievement of job performance. However, workplace autonomy is not a good predictor of lecturers’ job satisfaction. Therefore, lecturers require the appropriate directions and guidelines to understand the job requirements and achieve enjoyment and performance in a teaching job. The result demonstrated that the lecturer’s job satisfaction insignificantly boosted the lecturer’s job performance. Job satisfaction may not lead to job performance, according to the current finding, which contradicts Ong et al. (2020).’s conclusion that job satisfaction does not harness job performance. The study also found that lecturers with high self-efficacy have higher job satisfaction. The findings were consistent with those of Matos et al. (2021), who found that self-efficacy is a prerequisite for work performance. In the mediation analysis, the lecturer’s job satisfaction was found to insignificantly mediate the association between intrinsic and extrinsic incentive components and job performance. The moderation study indicated that self-efficacy regulated the connection between job satisfaction and job performance.

Finally, the ANN analysis was conducted to estimate job satisfaction and job performance factors. As a result, the ANN model for job satisfaction has proven that three significant factors instigating job satisfaction are FR, promotion, and CCT. The model for job performance also confirms that the three essential factors harnessing job performance are self-efficacy, promotion, and autonomy. The management of PHEIs and the Malaysian higher education ministry must monitor academicians’ satisfaction and performance to increase the quality of education in the Malaysian higher education sector. This initiative would help maintain the quality of education in Malaysia and contribute to the realization of the USD 1.5 billion mark by 2026, thereby achieving Malaysia’s ambition of becoming a center of educational excellence and competitive international education hub in Southeast Asia. HEIs in Malaysia are now expected to achieve higher academic excellence (Hussein and Al-Emami, 2016). Henceforward, it is also important for the HEIs to meet the international academic trends by raising the overall academic standards as well as the quality of the education.

Three key limitations highlighted in this study. First, the study only employed the limiting factors as the motivational factors to develop job satisfaction by harnessing job performance. Therefore, it is suggested that future studies incorporate more relevant motivational factors promoting job satisfaction and job performance, such as emotional intelligence, mindfulness, and other personal attributes. Second, job satisfaction in work settings also requires top management support and good relationships. However, this study only presented opinions about the limited personal factors that contribute to job satisfaction. The performance expectation should be formed with the mutual consent of the parties involved, while the execution of the job role requires support from the top management. Finally, the current research assumed a quantitative stance, which led to limited generalization and demonstration of the phenomenon under study. Thus, it is suggested that future research incorporates a mixed-method approach or multi-respondent (Lecturers-Deans) approach to understand the lecturers’ job satisfaction and performance.

## Data Availability Statement

The original contributions presented in the study are included in the article/Supplementary Material, further inquiries can be directed to the corresponding author.

## Ethics Statement

Ethical review and approval was not required for the study on human participants in accordance with the local legislation and institutional requirements. The patients/participants provided their written informed consent to participate in this study.

## Author Contributions

MM, ZM, and NH: conceptualization, instrument, data collection, and writing – original draft. SS and AA: conceptualization, data collection, data analysis, and writing – revision and amendments. All authors contributed to the article and approved the submitted version.

## Conflict of Interest

The authors declare that the research was conducted in the absence of any commercial or financial relationships that could be construed as a potential conflict of interest.

## Publisher’s Note

All claims expressed in this article are solely those of the authors and do not necessarily represent those of their affiliated organizations, or those of the publisher, the editors and the reviewers. Any product that may be evaluated in this article, or claim that may be made by its manufacturer, is not guaranteed or endorsed by the publisher.

## Footnotes

1
https://webpower.psychstat.org/wiki/tools/index
