# Supplementary material for: Modeling the Significance of Motivation on Job Satisfaction and Performance Among the Academicians: The Use of Hybrid Structural Equation Modeling-Artificial Neural Network Analysis
Source: Front Psychol. 2022 Jun 20;13:935822. doi: 10.3389/fpsyg.2022.935822 (PMC9251502; doi:10.3389/fpsyg.2022.935822)
Supplement: Supplementary file 2 [file Data_Sheet_2.pdf]

Supplementary Appendix 1. Study Population

No	Selection of respondents	No. of PHEIs
1	Universities (PHEIs)	51
2	International branch campuses	10
3	College universities	38
4	Colleges	349
	Total Population Size	448

Note: PHEI - Private Higher Educational Institutions

Supplementary Appendix 2. The selection of universities for this study

Zone	Name of PHEI	State
North	Universiti Teknologi Petronas (UTP)	Perak
	Universiti Tunku Abdul Rahman (UTAR)	
	Universiti Sultan Azlan Shah (USAS)	
	Quest International University	
	Universiti Terbuka Wawasan	Penang
	AIMST University	Kedah
	Universiti Kuala Lumpur Malaysian Spanish Institute (UNIKL- MSI)	
	Universiti Antarabangsa AlBukhary (AIU)	
Central	Multimedia University (MMU), Kampus Cyberjaya	Selangor
	Asia Pacific University of Technology & Innovation (Asia Pasific UTI)	
	UNITAR International University	
	Universiti Selangor (UNISEL), Kampus Shah Alam	
	Malaysia University of Sceince and Technology (MUST)	
	University Malaysia of Computer Science and Engineering	
	Al-Madinah International University (MEDIU)	
	City University	
	Universiti Teknologi Kreatif Limkokwing (LUCT)	
	Asia e University (AeU)	
	International Centre for Education in Islamic Finance (INCEIF)	
	Management and Science University (MSU)	
	Taylor's University	
	Manipal International University (MIU), Malaysia	
	Sunway University	
	Perdana University	
	Universiti HELP	
	Universiti SEGi (SEGi University)	
	Binary University of Management & Entrepreneurship (BUME)	
	Malaysia Institute For Supply Chain Innovation (MISI)	
	Universiti Metropolitan Asia (Asia Metropolitan University)	
	Universiti Tun Abdul Razak (UNIRAZAK)	Wilayah Persekutuan Kuala Lumpur
	Universiti Terbuka Malaysia (UNITEM) (Open University Malaysia (OUM)	
	Universiti Kuala Lumpur (UNIKL)	
	Universiti UCSI (UCSI University)	
	Global NXT University	
	Universiti Infrastruktur Kuala Lumpur	
	MAHSA University	
	International University of Malaya Wales (IUMW)	
	Universiti Kuala Lumpur Malaysian France Institute (UNIKL- MFI)	
	Universiti Kuala Lumpur Malaysian Institute of Aviation Technology (UNIKL-MIAT)	
	Universiti Kuala Lumpur British Malaysia Institute (Unikl-BMI)	
	Asia School Of Business (ASB)	
	Meritus University	
	Universiti Tenaga Nasional (UNITEN), Kampus Putrajaya	Wilayah Persekutuan Putrajaya
South	Raffles University Iskandar Malaysia	Johor
	Multimedia University (MMU), Kampus Melaka	Melaka
	Sekolah Klinikal Universiti Perubatan Antarabangsa (IMU)	Negeri Sembilan
	INTI International University	
	Universiti Nilai	
East Cost	Universiti Tenaga Nasional (UNITEN), Kampus Muadzam Shah	Pahang
	DRB-HICOM University Of Automotive Malaysia	
	Universiti UCSI (UCSI University), Kampus	Terengganu

	Terengganu	
Total		51

Supplementary Appendix 3. Survey Instrument

Code	Items	Sources
FR1	I am satisfied with my present financial rewards	(Chiang & (Shawn) Jang, 2008; Teclemichael Tessema & Soeters, 2006)
FR2	I am satisfied with my annual financial rewards	
FR3	My institution sets clear criteria for the overall reward system	
FR4	There is a well-established appraisal system in my institution	
FR5	My institution offers me an attractive compensation system	
FR6	The presence of financial reward reflects my current living standard	
PR1	Promotions are given based on my performance outcome	(Munyengabe et al., 2017b)
PR2	The way promotions are given motivates me to work hard.	
PR3	Incentives given are relevant compared to the work I do.	
PR4	Promotions in my institution are given based on merit to the most qualified	
PR5	My superior is fair in recommending promotions	
PR6	The promotion system is flexible in terms of weight given to teaching, research, and service length.	
PA1	Performance appraisal process is conducted in a regular periodic time (each semester)	(Al-Ashqar, 2017)
PA2	The performance appraisal system helps me to develop and improve my performance	
PA3	The students' evaluation process provides feedback on my teaching methods	
PA4	Performance appraisal helps me to know the strengths and weaknesses of my performance	
PA5	The performance appraisal process is characterized by the flexibility to face the changes in my teaching.	
PA6	Research, publication, conference participation included in my performance appraisal process.	
CE1	I have self-confident in my classroom management	(Munyengabe et al., 2017b)
CE2	The number of students doesn't affect my teaching activities in class	
CE3	I'm satisfied with the tidiness of my classroom	
CE4	Student's performance motivates my lecture activities	
CC1	The way the code of conduct is set doesn't interrupt me	(Munyengabe et al., 2017b)
CC2	I always obey the rules and regulations set by my institution	
CC3	I seek for colleague's permission when using their teaching material	
CC4	There is no injustice in handling issues related to my job performance	
AT1	I am free to decide how to go about getting my work done	(Johari et al., 2018)
AT2	I'm able to choose the way to go about my work in my team	
AT3	I am able to decide for myself what my objective are.	
AT4	I am free to choose how to carry out my work	
AT5	I have some control over what I suppose to do to achieve my goal	
AT6	I have the authority to decide on when to do particular activities as a part of my work	
JS1	I am satisfied with my current salary scheme	(Munyengabe et al., 2017b)
JS2	I am satisfied with my career prospect in this institution	
JS3	I am satisfied with my job permanency in this institution	
JS4	I am satisfied with the way my superior give feedback during appraisals.	
JS5	I am satisfied with the classroom which is equipped with all necessities to favour teaching activities.	
JS6	I am satisfied with my work because there is a clear policy towards educational aspirations.	
JS7	I am satisfied with the present social status as an academician.	
SE1	I feel confident in doing research	(Sharp et al., 2013)
SE2	I feel confident in journal publications	
SE3	I feel confident in delivering lecturing duties	
SE4	I feel confident updating lecture material constantly	
SE5	I feel confident in facilitating students' participation in class	
SE6	I feel confident in providing feedback on students' assessment	
JP1	I adequately accomplish the tasks given on time	(Molefe, 2010; Sukirno & Siengthai, 2011)
JP2	I fulfill the responsibilities specified in my job description	
JP3	I engaged in activities that will directly affect my job performance evaluation	
JP4	I provide an alternative solution for the problem encountered by the institution	
JP5	I have relevant knowledge with my current position	
JP6	I respond appropriately to my superior's feedback on my job performance	

Note: Financial Rewards (FR), Promotion (PR), Performance Appraisals (PA), Classroom Environment (CE), Code of Conduct (CC), Autonomy (AT), Job Satisfaction (LS), Self-Efficacy (SE), Job Performance (JP)
Source: Author's data analysis

Supplementary Appendix 4. Loadings and Cross-Loading

Code	FR	PR	PA	CE	CC	AT	JS	SE	JP
FR1	0.868	0.616	0.489	0.250	0.322	0.229	0.656	0.214	0.191
FR2	0.851	0.625	0.505	0.209	0.333	0.222	0.635	0.184	0.192
FR3	0.812	0.683	0.569	0.178	0.362	0.372	0.633	0.212	0.233
FR4	0.788	0.648	0.565	0.180	0.304	0.370	0.573	0.226	0.279
FR5	0.824	0.654	0.538	0.186	0.358	0.272	0.598	0.148	0.171
FR6	0.720	0.469	0.427	0.268	0.305	0.339	0.510	0.245	0.229
PR1	0.640	0.871	0.609	0.190	0.380	0.350	0.627	0.213	0.308
PR2	0.627	0.871	0.624	0.209	0.365	0.342	0.640	0.195	0.277
PR3	0.750	0.849	0.590	0.186	0.360	0.322	0.653	0.203	0.239
PR4	0.641	0.855	0.599	0.195	0.333	0.361	0.634	0.185	0.241
PR5	0.506	0.759	0.483	0.157	0.363	0.323	0.560	0.158	0.195
PR6	0.631	0.787	0.516	0.097	0.289	0.264	0.599	0.097	0.119
PA1	0.472	0.452	0.666	0.205	0.399	0.251	0.447	0.215	0.253
PA2	0.625	0.691	0.849	0.196	0.356	0.378	0.617	0.183	0.273
PA3	0.317	0.376	0.658	0.314	0.287	0.280	0.402	0.282	0.288
PA4	0.506	0.552	0.862	0.253	0.292	0.355	0.540	0.266	0.289
PA5	0.526	0.560	0.820	0.215	0.340	0.334	0.561	0.235	0.264
PA6	0.351	0.388	0.580	0.229	0.300	0.283	0.427	0.266	0.238
CE1	0.142	0.072	0.210	0.824	0.456	0.359	0.304	0.643	0.548
CE2	0.167	0.126	0.159	0.681	0.359	0.271	0.278	0.358	0.342
CE3	0.275	0.248	0.279	0.753	0.436	0.302	0.401	0.459	0.408
CE4	0.184	0.177	0.257	0.675	0.404	0.343	0.244	0.456	0.459
CC1	0.340	0.285	0.345	0.421	0.746	0.368	0.511	0.409	0.428
CC2	0.234	0.248	0.227	0.492	0.759	0.324	0.377	0.498	0.459
CC3	0.230	0.230	0.277	0.431	0.706	0.424	0.318	0.451	0.442
CC4	0.374	0.459	0.427	0.298	0.697	0.376	0.490	0.239	0.303
AT1	0.247	0.273	0.283	0.364	0.407	0.847	0.363	0.323	0.361
AT2	0.302	0.313	0.364	0.370	0.448	0.837	0.402	0.393	0.418
AT3	0.310	0.330	0.355	0.337	0.430	0.842	0.422	0.376	0.434
AT4	0.292	0.309	0.338	0.336	0.401	0.876	0.366	0.300	0.380
AT5	0.346	0.355	0.380	0.417	0.451	0.862	0.437	0.424	0.473
AT6	0.360	0.406	0.424	0.387	0.456	0.842	0.460	0.408	0.450
JS1	0.776	0.620	0.508	0.215	0.338	0.280	0.739	0.178	0.213
JS2	0.653	0.683	0.584	0.258	0.463	0.389	0.826	0.250	0.293
JS3	0.514	0.530	0.495	0.308	0.438	0.404	0.727	0.371	0.335
JS4	0.521	0.611	0.584	0.260	0.489	0.359	0.745	0.291	0.324
JS5	0.349	0.383	0.359	0.442	0.447	0.292	0.625	0.393	0.390
JS6	0.584	0.591	0.547	0.335	0.481	0.419	0.827	0.294	0.329
JS7	0.482	0.467	0.462	0.413	0.443	0.395	0.759	0.469	0.443
SE1	0.225	0.206	0.260	0.393	0.355	0.325	0.338	0.704	0.512
SE2	0.211	0.149	0.224	0.322	0.315	0.259	0.289	0.630	0.421
SE3	0.162	0.136	0.219	0.652	0.507	0.358	0.343	0.904	0.708
SE4	0.222	0.179	0.285	0.588	0.498	0.439	0.370	0.895	0.707
SE5	0.231	0.203	0.304	0.660	0.523	0.388	0.388	0.887	0.713
SE6	0.212	0.186	0.278	0.568	0.466	0.380	0.357	0.869	0.695
JP1	0.195	0.186	0.226	0.486	0.479	0.404	0.325	0.678	0.864
JP2	0.170	0.193	0.240	0.505	0.476	0.447	0.341	0.688	0.895
JP3	0.247	0.268	0.370	0.490	0.470	0.411	0.393	0.583	0.824
JP4	0.198	0.221	0.251	0.428	0.386	0.380	0.325	0.528	0.713
JP5	0.222	0.216	0.301	0.608	0.507	0.431	0.373	0.756	0.866
JP6	0.304	0.328	0.410	0.497	0.487	0.418	0.447	0.628	0.842

Note: Financial Rewards (FR), Promotion (PR), Performance Appraisals (PA), Classroom Environment (CE), Code of Conduct (CC), Autonomy (AT), Job Satisfaction (LS), Self-Efficacy (SE), Job Performance (JP)
Source: Author's data analysis
